# Improved production of 2,3‐butanediol and isobutanol by engineering electron transport chain in *Escherichia coli*

**DOI:** 10.1111/1751-7915.13669

**Published:** 2020-09-20

**Authors:** Hwi‐Min Jung, Jae‐Ho Han, Min‐Kyu Oh

**Affiliations:** ^1^ Department of Chemical and Biological Engineering Korea University 145 Anam‐ro, Seongbuk‐gu Seoul 02841 Korea

## Abstract

The electron transport chain (ETC) is one of the major energy generation pathways in microorganisms under aerobic condition. Higher yield of ATP can be achieved through oxidative phosphorylation with consumption of NADH than with substrate level phosphorylation. However, most value‐added metabolites are in an electrochemically reduced state, which requires reducing equivalent NADH as a cofactor. Therefore, optimal production of value‐added metabolites should be balanced with ETC in terms of energy production. In this study, we attempted to reduce the activity of ETC to secure availability of NADH. The ETC mutants exhibited poor growth rate and production of fermentative metabolites compared to parental strain. Introduction of heterologous pathways for synthesis of 2,3‐butanediol and isobutanol to ETC mutants resulted in increased titres and yields of the metabolites. ETC mutants yielded higher NADH/NAD^+^ ratio but similar ATP content than that by the parental strain. Furthermore, ETC mutants operated fermentative metabolism pathways independent of oxygen supply in large‐scale fermenter, resulting in increased yield and titre of 2,3‐butanediol. Thus, engineering of ETC is a useful metabolic engineering approach for production of reduced metabolites.

## Graphical abstract

In this study, we attempted to reduce the electron transport chain (ETC) activity by making multiple mutants to secure availability of NADH, and then heterologous pathways for synthesis of 2,3‐butanediol and isobutanol were introduced. ETC mutants produced higher levels of metabolites with higher NADH/NAD^+^ ratio. The results proved that engineering of ETC is a useful metabolic engineering approach for production of reduced metabolites.

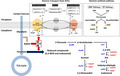

## Introduction

Most of the facultative aerobes catabolize carbon sources through glycolysis and TCA cycle and generate additional ATP through respiration under aerobic conditions, which is called oxidative phosphorylation. It is known that the cellular energy production from oxidative phosphorylation (OXPHO) is a higher‐energy‐yield metabolic process than substrate level phosphorylation (SLPHO) via fermentative metabolism (Ward, 2015). Therefore, better fitness of host strain is usually observed under aerobic conditions. This is advantageous to operation of industrial bioprocesses because high cell density cultivation of host strain results in increased productivity during the bioprocess operation. However, oxidized intracellular redox state is observed under aerobic conditions. Reduced condition is often required for the production of value‐added compounds such as biofuels and commodity chemicals. Cellular reducing cofactors such as NAD(H) are involved in the intracellular redox reaction. Therefore, most bioprocesses are frequently operated in microaerobic conditions, and fine optimization is inevitable to satisfy both cell growth and reducing power (Wong *et al*., 2014; Chan *et al*., 2016; Wu *et al*., 2016). An alternative approach involving the separation of growth phase and production phase, by manipulating oxygen supply, has been attempted for efficient production of target metabolites (Lee *et al*., 2019). Although several different methods exploring these aspects of the process control have been suggested, problems about inhomogeneous oxygen level in large‐scale fermenter and discontinuous oxygen supply sometimes disable the metabolism of host strain (Chubukov *et al*., 2016).

Recently, a different perspective regarding the efficiency of SLPHO and OXPHO has been suggested in several studies (Chen and Nielsen, 2019; Basan *et al*., 2015; Jung *et al*., 2019). Although higher amount of ATP is generated via OXPHO than SLPHO, higher protein costs are required to operate OXPHO. According to a previous study, SLPHO has advantages such as high ATP production rates, which results in increased growth rate and glucose uptake rate (Basan *et al*., 2015). Some microorganisms exhibit active fermentative pathway despite the presence of oxygen. *Saccharomyces cerevisiae*, for example, mainly produced a fermentative product, ethanol, using large amounts of glucose under aerobic conditions, which is called Crabtree effect (Pfeiffer and Morley, 2014). Similar metabolic characteristics have been observed in various organisms in substrate rich conditions [lactic acid production in cancer cell; succinate production in trypanosomatids; 2,3‐butanediol (2,3‐BDO) production in *Klebsiella pneumoniae* and *Enterobacter aerogenes*] (Bringaud *et al*., 2010; Jung *et al*., 2014; Ji *et al*., 2011; Goodwin *et al*., 2015). Microorganisms have evolved to have an active glycolysis and fermentative pathway rather than TCA cycle and respiration to rapidly take up carbon sources from the surroundings. Mimicking these characteristics makes it possible to improve sugar consumption and enhance the production of reduced metabolites under aerobic condition in *Escherichia coli*.

The electron transport chain (ETC) complex is a significant workhorse for bacterial respiration under aerobic conditions. The oxidation of electron donors and reduction of electron acceptors generates electron flow, and it drives the proton motive force, using which intracellular ATP can be generated. Under aerobic conditions, NADH and oxygen were utilized as electron donor and acceptor respectively. The electron from oxidation of NADH is transferred to oxygen through ETC components forming H_2_O. The ETC of *E. coli* is composed of two protein complexes, namely NADH dehydrogenase, and terminal oxidase, in which the electron transfer is mediated by membrane quinone (Sousa *et al*., 2012). The cellular ATP can be synthesized by ATPase using the proton gradient generated by ETC, which is called oxidative phosphorylation. There are two major NADH dehydrogenases in *E. coli*. The first being NADH dehydrogenase I (NDH‐1, encoded by *nuo* operon), which is composed of 13 protein subunits, generating proton motive force, H^+^/e^−^ = 2, during electron transfer to quinone pool. The other being NADH dehydrogenase‐2 (NDH‐2, encoded by *ndh*), which is a single unit enzyme that is not engaged in proton motive force (H^+^/e^−^ = 0) but in intracellular redox management (Matsushita *et al*., 1987). There are several types of membrane quinols, such as demethylmenaquinol (DMK), menaquinol (MK) and ubiquinol (UQ), in *E. coli*. The expression of ubiquinone synthesis pathway was repressed by global regulators such as Fnr and ArcA under anaerobic conditions (van Beilen and Hellingwerf, 2016). Generally, ubiquinol is dominantly synthesized under aerobic conditions and menaquinol under anaerobic conditions. The three terminal oxidases, namely CyoABCD (H^+^/e^−^ = 2), CydAB (H^+^/e^−^ = 1) and CbdAB (H^+^/e^−^ = 0), are functional in *E. coli* (Puustinen *et al*., 1991). The Cyo and Cyd complex can generate proton motive force, but the role of Cbd complex is not fully known. All these components can be candidates for engineering to modulate the respiration activity of *E. coli*.

The metabolic changes owing to mutations of ETC components have been demonstrated in various bacteria such as *E. coli*, *Pseudomonas* sp., *Corynebacterium glutamicum*, *Bacillus subtilis*, *Lactococcus lactis and Zymomonas mobilis* (Nies *et al*., 2019; Koch‐Koerfges *et al*., 2013; Zhu *et al*., 2011; Kalnenieks *et al*., 2019; Wu *et al*., 2015). In most of the cases, the manipulation of respiration level resulted in retardation of growth, decreased oxygen uptake and improved production of organic compounds, such as lactate, acetate, succinate and ethanol, under aerobic condition. Especially, in the case of *B. subtilis*, the ETC mutant exhibited better fitness than that of the wild‐type strain because of the inefficient function of ETC, which contributes to enhanced production of target metabolites such as riboflavin and *N‐*acetylglucosamine (Zamboni *et al*., 2003; Liu *et al*., 2014). In a different approach, the activity of ETC was controlled by engineering membrane lipid composition of *E. coli*, which affected the diffusivity of membrane quinon(ol) (Budin *et al*., 2018).

In this study, the activity of ETC was controlled to produce reduced compounds under aerobic conditions. The expression of ETC component proteins (NADH dehydrogenase, membrane quinone and terminal oxidase) was manipulated by using CRISPR/Cas9 system to achieve a highly reduced intracellular state in *E. coli*. The resulting strains exhibited the phenotype of aerobic fermentation with accumulated pyruvate and acetate. When the synthetic pathway for 2,3‐BDO and isobutanol was introduced to the mutants, the production of these metabolites was highly improved, regardless of oxygen supply. The intracellular concentration of NADH and ATP was analysed to understand their effects on the production of reduced compounds. The oxygen‐insensitive metabolic activities were observed in ETC mutant, which were crucial to large‐scale fermentation. It was confirmed that formulation of reduced state is helpful for production of 2,3‐BDO and isobutanol in ETC mutants.

## Results and discussion

### Phenotypical changes of ETC mutation

**Fig. 1:**
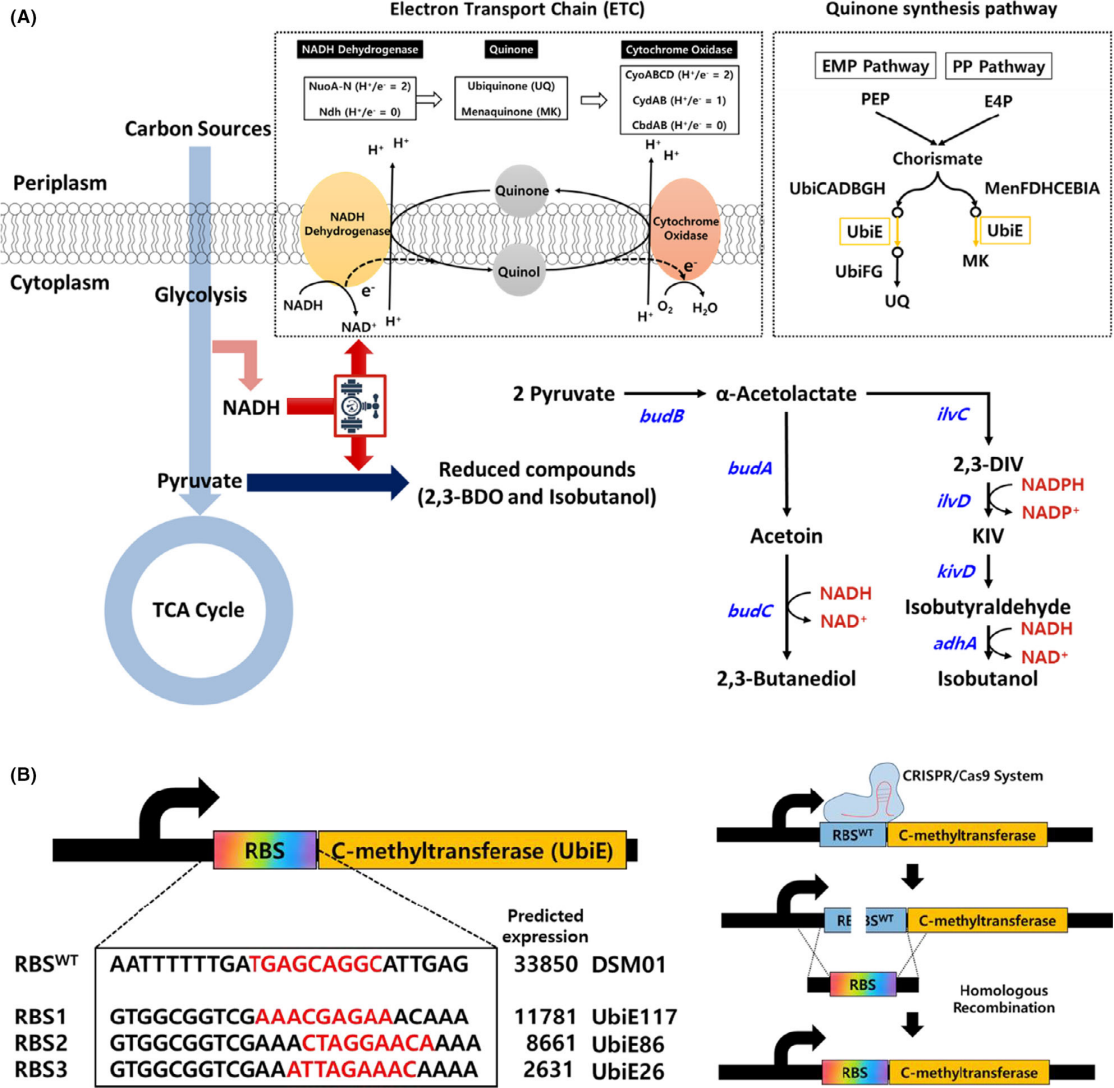
(A) The overall pathway scheme of electron transport chain in engineered mutants with heterologous gene expression for synthesis of reduced compounds. The redox cofactor NADH is generated by glycolysis and TCA cycle and consumed by electron transport chain under aerobic conditions. The intracellular NADH can be accumulated when the activity of electron transport chain was inactivated despite aerobic conditions. The two NADH dehydrogenases (Nuo and Ndh) and two terminal oxidases (Cyo and Cyd) were removed in combinations, and expression of quinone synthesis pathway (*ubiE*) was modulated in this study, resulting in the construction of seven ETC mutants. The heterologous pathways were expressed using the electron transport chain inactivated mutants as host strains to produce reduced compounds. The overexpressed heterologous genes are marked in blue (*budB*, acetolactate synthase; *budA*, acetolactate decarboxylase; *budC*, acetoin reductase; *ilvC*, ketol‐acid reductoisomerase; *ilvD*, dihydroxyacid dehydratase; *kivD*, α‐ketoisovalerate decarboxylase; and *adhA*, alcohol dehydrogenase). The required cofactors for synthesis of reduced compounds such as 2,3‐butanediol and isobutanol are marked in red. Several metabolites were written in abbreviation (2,3‐DIV, 2,3‐dihydroxyisovalerate; KIV, 2‐ketoisovalerate; UQ, Ubiquinone; and MK, Menaquinone). (B) Modification of *ubiE* 5′‐UTR using CRISPR/Cas system. The expression levels of UbiE in wild‐type strain were predicted and synthetic UTR sequences which are expected to have lower expression levels were designed by UTR designer. The RBS of *ubiE* was replaced using CRISPR/Cas9 guided cleavage followed by the repair of the broken DNA via homologous recombination. As a result, three engineered strains were constructed (UbiE117, UbiE86 and UbiE26).

The ETC is one of the major energy generating systems in *E. coli* under aerobic condition. The NADH supplied from glycolysis and TCA cycle can be oxidized by ETC, during which cellular ATP can be produced. When the ETC of a strain is inactivated, the metabolism of the mutant is similar to that observed under anaerobic conditions for the parent strain. The ETC of *E. coli* works through three components, namely NADH dehydrogenase, quinone, and terminal oxidase, using NADH as the electron donor and oxygen as the electron acceptor (Fig. 1). The phenotypical changes of mutants with inactivated ETC components were investigated in this study. As shown in Table 1, the two NADH dehydrogenase (Nuo and Ndh) were inactivated in combination with the two terminal oxidases (Cyo and Cyd), thus, forming four mutants (ETC1, ETC2, ETC3 and ETC4). Cbd, one of the three terminal oxidases, was excluded due to its negligible effect on respiration (Bekker *et al*., 2009). The membrane quinone synthesis pathway was also modulated to reduce the ETC activity. The expression of UbiE (demethylmenaquinone methyltransferase/2‐methoxy‐6‐polyprenyl‐1,4‐benzoquinol methylase), which is involved in both ubiquinone and menaquinone synthesis pathway of *E. coli* (Fig. 1A), was controlled by engineering its 5′‐untranslated region (UTR) (Ravcheev and Thiele, 2016). The native expression level of *ubiE* was predicted by bioinformatics web tool UTR designer and three artificial UTRs having lower expression levels were rationally designed (Fig. 1B). The three artificial UTRs were inserted to genomic DNA of parental strain DSM01 using CRISPR/Cas9 system (UbiE117, UbiE86, and UbiE26). For targeting 5′‐UTR of *ubiE*, CRISPR RNA‐expression plasmid (pZS CRISPR *ubiE* RBS sacB) and Cas9‐expression plasmid (pCas9) were introduced into the parental strain (Table 1). The three types of double‐stranded linear DNA were introduced with homologous recombination for repair of Cas‐mediated cleavage (Fig. 1B). The modified UTR of *ubiE* was confirmed by sequence analysis (Fig. S2).

**Table 1:** Bacterial strains and plasmids used in this study.

Strains	Descriptions	References
DSM01	MG1655(DE3)*△frdA::FRT△pta::FRT△ldhA::FRT△adhE::FRT*	Baek and colleagues (2013)
ETC1	DSM01 *△ndh △cyd △cbd*	This study
ETC2	DSM01 *△ndh △cyo △cbd*	This study
ETC3	DSM01 *△nuo △cyd △cbd*	This study
ETC4	DSM01 *△nuo △cyo △cbd*	This study
UbiE117	DSM01 with 34% *ubiE* expression expected	This study
UbiE86	DSM01 with 25% *ubiE* expression expected	This study
UbiE26	DSM01 with 7% *ubiE* expression expected	This study
DSM01‐BDO	DSM01 harbouring pZS‐BDO	This study
ETC1‐BDO	ETC1 harbouring pZS‐BDO	This study
ETC2‐BDO	ETC2 harbouring pZS‐BDO	This study
ETC3‐BDO	ETC3 harbouring pZS‐BDO	This study
ETC4‐BDO	ETC4 harbouring pZS‐BDO	This study
UbiE117‐BDO	UbiE117 harbouring pZS‐BDO	This study
UbiE86‐BDO	UbiE86 harbouring pZS‐BDO	This study
UbiE26‐BDO	UbiE26 harbouring pZS‐BDO	This study
DSM01‐IB	DSM01 harbouring pZA‐DCB and pBT‐DA	This study
ETC1‐IB	ETC1 harbouring pZA‐DCB and pBT‐DA	This study
ETC2‐IB	ETC2 harbouring pZA‐DCB and pBT‐DA	This study
ETC3‐IB	ETC3 harbouring pZA‐DCB and pBT‐DA	This study
ETC4‐IB	ETC4 harbouring pZA‐DCB and pBT‐DA	This study
UbiE117‐IB	UbiE117 harbouring pZA‐DCB and pBT‐DA	This study
UbiE86‐IB	UbiE86 harbouring pZA‐DCB and pBT‐DA	This study
UbiE26‐IB	UbiE26 harbouring pZA‐DCB and pBT‐DA	This study

Plasmids	Descriptions	References
pKD46	Red recombinase expression vector, AmpR	Gene bridge
707 FLP	Flippase expression plasmid, pSC101ori, *repA*, cl578, FLPe, TetR	Gene bridge
pCas9	Cas9 nuclease, crRNA, tracrRNA, CmR	Jiang and colleagues (2013)
pZS CRISPR SacB	pZS21MCS, but TracerRNA‐crRNA‐P_sacB::_ *sacB*	Heo and colleagues (2017)
pZS CRISPR *ubiE* RBS SacB	pZS CRISPR with *ubiE* RBS targeting crRNA	This study
pZS‐BDO	*budABC* from *E. aerogenes* KCTC2190 in pZS21 MCS	Mazumdar and colleagues (2013)
pZADCB	*ilvD*, *ilvC* and *budB* from *K. pneumoniae* KCTC2242 in pZA31 MCS	Jung and colleagues (2017)
pBTDA	*kivD* and *adhA* from *L. lactis* Il1403 in pBTBX‐2	Jung and colleagues (2017)

**Fig. 2:**
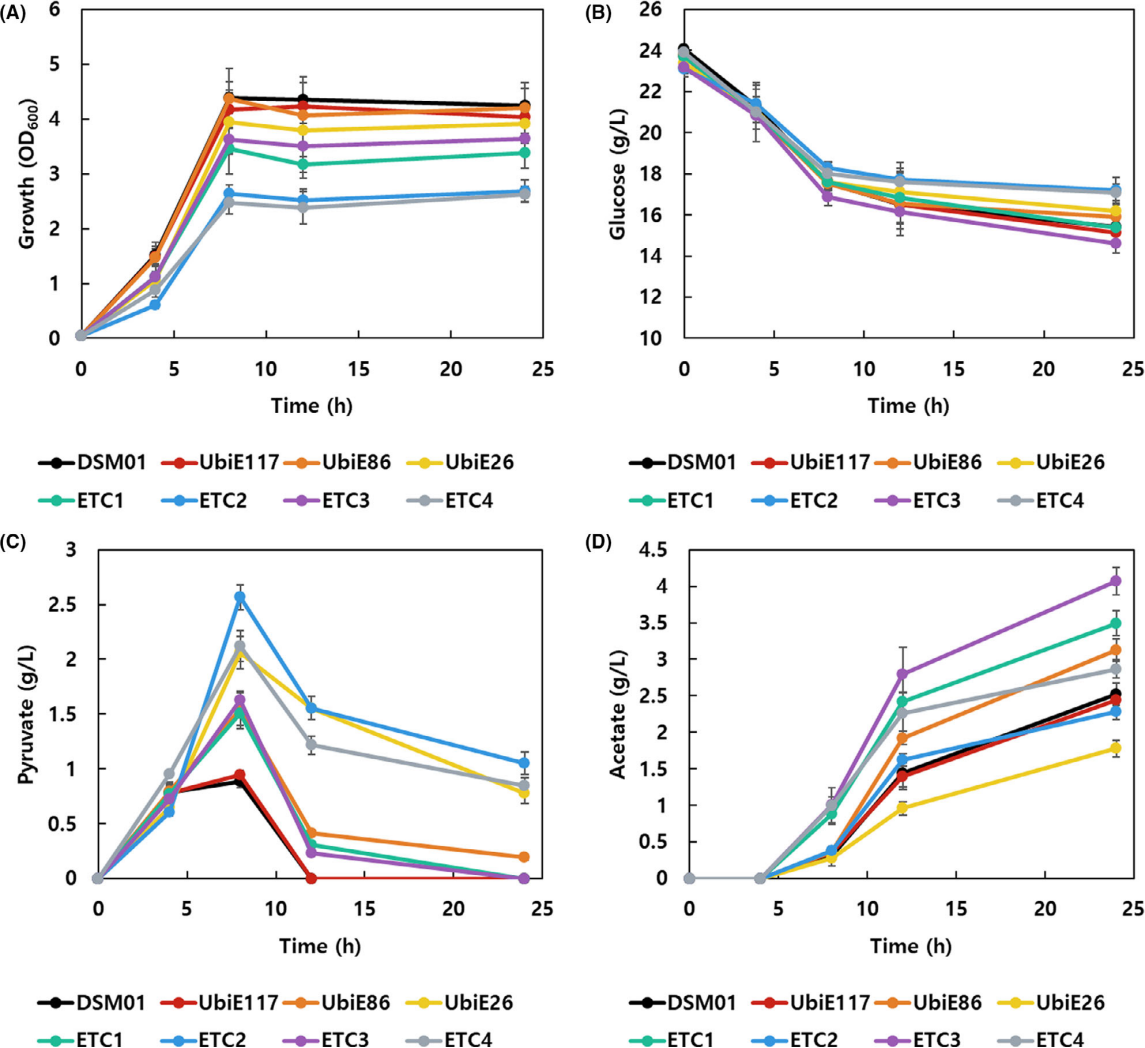
The growth and metabolite production in parental strain and ETC mutants. (A) The growth profiles, (B) glucose consumption, (C) pyruvate production, and (D) Acetate accumulation of DSM01 and ETC mutants are shown for 24 h flask cultivation. The ETC mutants exhibited retarded growth, reduced glucose consumption, and a higher accumulation of pyruvate and acetate compared to the parental strain DSM01.

The seven constructed mutants and parental strain were cultivated in flask. The growth retardation of all mutant strains was identified. The UbiE knockdown mutants exhibited 2–8% lower final OD, while the oxidoreductase mutants (ETC1, ETC2, ETC3 and ETC4) exhibited 15–39% lower final OD (Fig. 2A). These results corresponded to previous reports that inactivation of ETC inhibited growth of the host strain (Zambrano and Kolter, 1993; Prüss *et al*., 1994). The glucose uptake of ETC mutants was equal or higher, while their fitness was impeded compared to the parental strain (Fig. 2B). The improved glucose uptake in non‐respirating *E. coli* has been well discussed in previous reports (Gonzalez *et al*., 2017; Chen *et al*., 2011). Interestingly, 6–190% higher accumulation of pyruvate was observed in ETC mutants in the early stationary phase. The pyruvate was completely re‐consumed in the parental strain DSM01, but excessive pyruvate was left at the end of fermentation in several ETC mutants (Fig. 2C). Furthermore, higher production of acetate was exhibited in most of the ETC mutants. The increased yields of acetate (32–63%) were seen in UbiE86, ETC1, ETC2, ETC3 and ETC4 after 24 h cultivation (Fig. 2D).

A previous report indicates that enzyme reactions involved in glycolysis, such as the function of the pyruvate dehydrogenase complex (PDHc), can be inhibited by a high redox ratio commonly observed in anaerobic conditions (Kim *et al*., 2008). Therefore, the high redox state of ETC mutants hinders the conversion of pyruvate to acetyl‐CoA, and excessive pyruvate is accumulated in the culture medium. Acetate is another major by‐product of ETC mutants. Acetate can be produced through pyruvate oxidase (PoxB)‐mediated pathway because the Pta and Ack‐mediated pathway had already been eliminated in DSM01. PoxB is a membrane‐bound enzyme that oxidizes pyruvate to acetate, which induces proton motive force by transferring electrons to the ETC module (Abdel‐Hamid *et al*., 2001). Therefore, the production of acetate can facilitate energy replenishment by consuming pyruvate. Furthermore, it has been demonstrated that the deletion of ETC components, especially quinone synthesis pathway and terminal oxidase, can increase the formation of superoxide ions (Korshunov and Imlay, 2006). This can be another reason for the aggravation of cellular growth. The pyruvate oxidation through PoxB might be favourable because it can divert the reaction of pyruvate dehydrogenase, which avoids NADH‐mediated electron transport system (Moreau, 2004). Accordingly, NADH‐consuming synthetic pathway utilizing pyruvate as a precursor could be effectively working in the ETC mutants.

### Improved production of 2,3‐butanediol (2,3‐BDO) and isobutanol in ETC mutants

We expected that the alteration of cellular metabolism observed in ETC mutants can have a positive effect on the production of reduced compounds. For preliminary experiments, the growth of DSM01 harbouring 2,3‐BDO and isobutanol pathway and production of reduced compounds were investigated in zinc ion containing culture medium, referring to the report that zinc ion inhibits activity of NADH dehydrogenase in *E. coli* (Schulte *et al*., 2014). Approximately 0–2 mM of ZnSO_4_ was added in the culture medium. The addition of zinc ion inhibited growth of strains; the strains hardly grew in the presence of 2 mM of zinc ion (Fig. S1A,D). We attempted to produce two reduced chemical products, 2,3‐BDO and isobutanol, from engineered strains since the redundant pyruvate was accumulated and higher redox ratio was expected in the ETC mutants. The 2,3‐BDO synthesis pathway was constructed by introducing *budABC* (from *E. aerogenes*) harbouring plasmid and the isobutanol production pathway by introducing plasmid harbouring *ilvDC and budB* (from *K. pneumoniae*) along with *kivD and adhA* (from *L. lactis*), to the engineered strains (Mazumdar *et al*., 2013; Jung *et al*., 2017). The 2,3‐BDO and isobutanol producing transformants were termed as ‘strain‐BDO’ and ‘strain‐IB’ respectively (Table 1). The production of isobutanol was gradually enhanced as the concentration of ZnSO_4_ increased. However, production of 2,3‐BDO exhibited negligible difference regardless of the addition of zinc ion (Fig. S1B,E). However, the specific yields of 2,3‐BDO and isobutanol improved up to 53% and 174%, respectively, in 12 h cultivation (Fig. S1C,F). These results denoted that inhibition of NADH oxidation was helpful for the production reduced chemical compounds in *E. coli*.

**Fig. 3:**
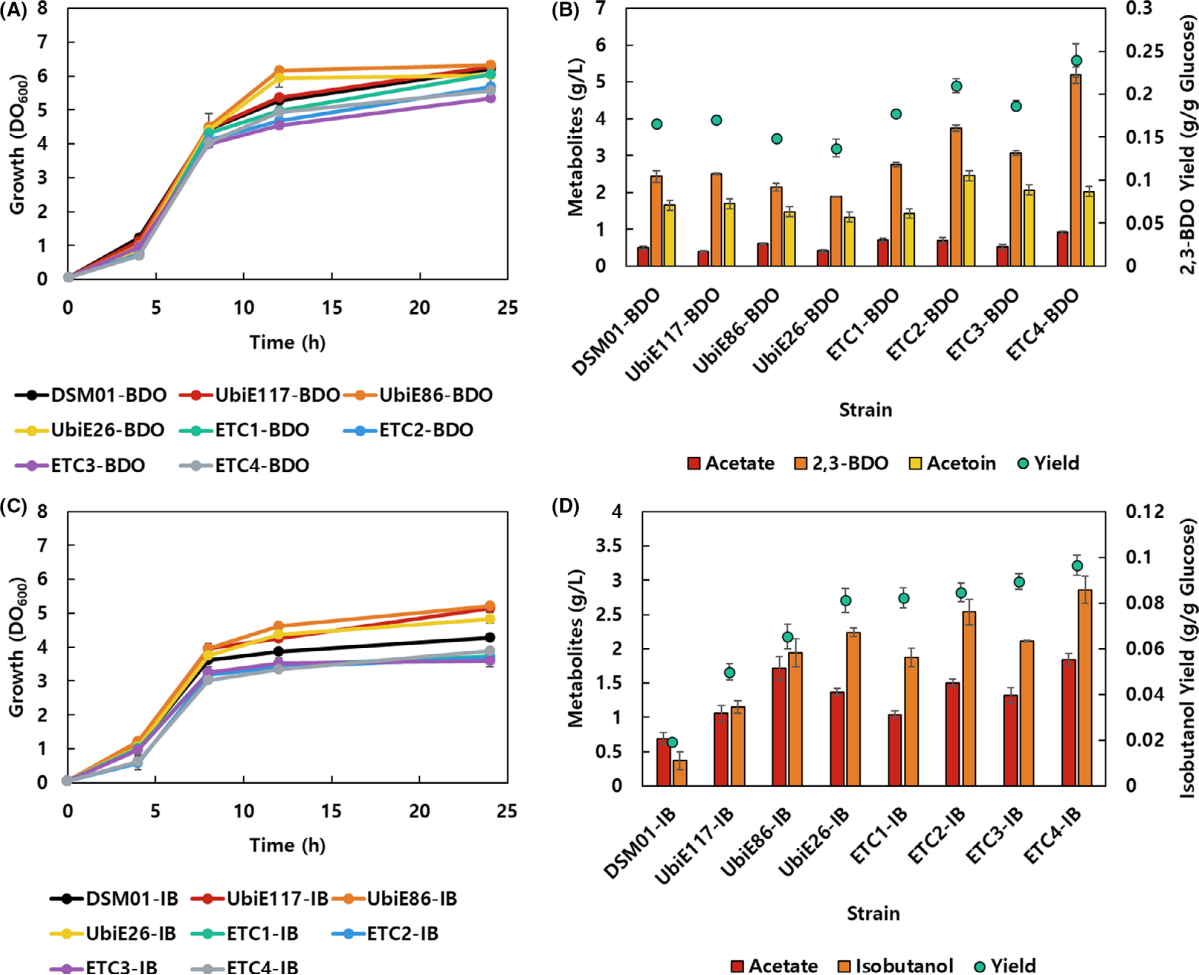
The growth and metabolite production in transformants engineered to produce reduced compounds. (A) Growth profiles for 24 h and (B) metabolites production at 24 h for 2,3‐butanediol‐producing transformants are shown. (C) Growth profiles for 24 h and (D) metabolite production at 24 h for isobutanol producing transformants are shown.

The growth deviation between parental strain and mutants was alleviated when the reduced compound synthesis pathway was expressed (Fig. 3A,C). We postulated that intracellular unused pyruvate and NADH were consumed by the introduced pathways, which relieved metabolic imbalance inside the cell. Therefore, the recovered growth was exhibited, and pyruvate was not produced at all by the strains with the introduced metabolic pathway (data not shown). The production of 2,3‐BDO was improved in ETC mutants, but was not in the UbiE knockdown mutants. The titres and yields were increased 13–113% and 6–44%, respectively, in four oxidoreductase mutants (Fig. 3B). Among the oxidoreductase mutants, Cyo mutants (ETC2‐BDO and ETC4‐BDO) exhibited higher production of 2,3‐BDO compared with Cyd mutants (ETC1‐BDO and ETC3‐BDO). Furthermore, the Nuo mutants (ETC3‐BDO and ETC4‐BDO) displayed higher production than Ndh mutants (ETC1‐BDO and ETC2‐BDO). Especially, the highest titre and yield of 2,3‐BDO were observed in ETC4‐BDO strain, which were 113% and 44%, respectively, more than those in DSM01‐BDO (Fig. 3B). On the other hand, the production of isobutanol was significantly improved in all ETC mutants and UbiE knockdown mutants. A gradual increase of isobutanol titre was observed in UbiE117‐IB, UbiE86‐IB and UbiE26‐IB, by 3.1‐fold, 5.2‐fold and 6.1‐fold, respectively, compared with the isobutanol titre in DSM01‐IB (Fig. 3D). The isobutanol production increased by 5.1‐fold, 6.9‐fold, 5.74‐fold and 7.7‐fold in ETC1‐IB, ETC2‐IB, ETC3‐IB and ETC4‐IB respectively (Fig. 3D). Similar to 2,3‐BDO production case, Cyo mutants (ETC2‐IB and ETC4‐IB) exhibited more effective production of isobutanol with respect to Cyd mutants (ETC1‐IB and ETC3‐IB). Also, Nuo mutants (ETC3‐IB and ETC4‐IB) were more effective for isobutanol production than Ndh mutants (ETC1‐IB and ETC2‐IB). Higher yields of isobutanol were confirmed in all ETC mutants than those in the parental strain, (DSM01‐IB) especially a 5.0‐fold increase was observed in ETC4‐IB strain (Fig. 3D). It is postulated that Nuo and Cyo are more influential to cell physiology and metabolism than Ndh and Cyd in this culture condition, which led to a synergetic effect for production of reduced compounds.

These results were consistent with previous studies that showed that inactivation of NADH dehydrogenases improved the production of 2,3‐BDO and isobutanol in *Klebsiella pneumoniae* and *Corynebacterium glutamicum* (Koch‐Koerfges *et al*., 2013; Zhang *et al*., 2018). It was expected that the improved production of the reduced compounds can be obtained by an increased intracellular NADH/NAD^+^ ratio induced from the inactivation of ETC. Because isobutanol is a chemically more reduced compound than 2,3‐BDO, isobutanol production pathway requires more reducing power (1 NADH and 1 NADPH from pyruvate) than the 2,3‐BDO production pathway (1 NADH). Thus, the intracellular redox ratio affected the production of isobutanol to a comparatively larger extent than 2,3‐BDO in the ETC mutants.

### Analysis of intracellular redox and energy state in ETC mutants

**Fig. 4:**
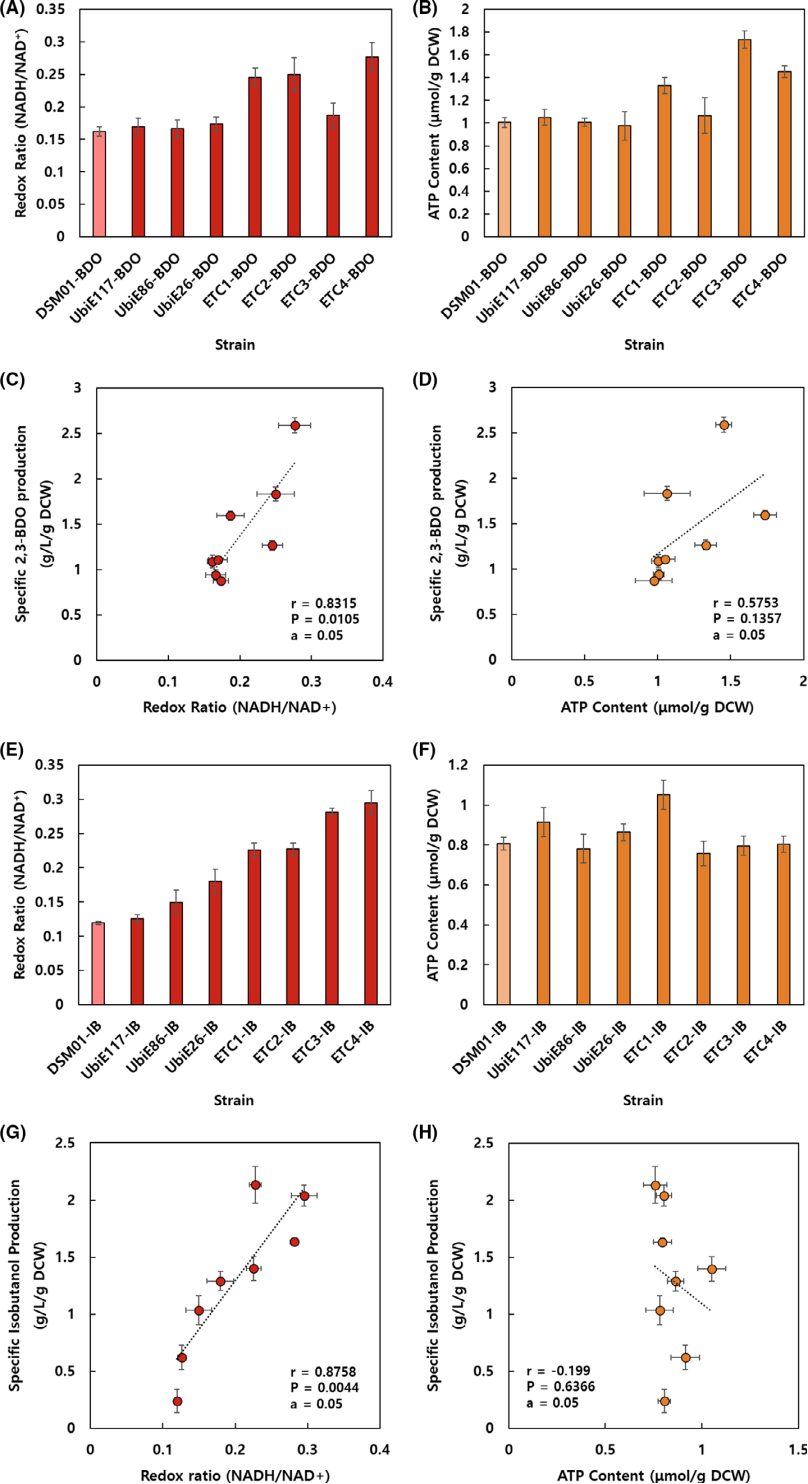
Intracellular redox ratio and ATP content on biomass. (A) Redox ratio and (B) ATP content of 2,3‐butanediol‐producing transformants are presented. Pearson correlations of specific 2,3‐butandiol titres and (C) redox ratios and (D) ATP contents are shown in graphs. Pearson correlations of specific isobutanol titres and (G) redox ratios and (H) ATP contents are shown in graphs. The Pearson correlation (*r*), *P* value (*P*) and significance level (*α*) was displayed in the graphs.

To comprehend the cause of metabolic alteration in ETC mutants, the intracellular redox and energy state were analysed. We tried to compare the NAD(H) and ATP contents of seven ETC mutants and parental strain harbouring reduced compound synthesis pathway. The culture broth was acquired from the middle of exponential phase in which the overall metabolic process could be vigorous involving central carbon metabolism and respiration. The redox ratios of UbiE knockdown mutants were comparable to that of the parental strain, which were in consistence with 2,3‐BDO production (Fig. 4A). However, the NADH/NAD^+^ increased by 52%, 55%, 16% and 71% in ETC1‐BDO, ETC2‐BDO, ETC3‐BDO and ETC4‐BDO, respectively, compared with the NADH/NAD^+^ in DSM01‐BDO (Fig. 4A). The ratios were especially high in Cyo mutants (ETC2‐IB and ETC4‐IB), explaining the improved production of 2,3‐BDO in those mutants (Fig. 4A). Considering the all UbiE and ETC mutants, we could confirm the positive correlation between redox ratio and 2,3‐BDO titres (Fig. 4C). Meanwhile, the ATP contents were similar between UbiE knockdown mutants compared to the parental strain, and were higher in Cyd mutants (ETC1‐IB and ETC3‐IB) and Nuo mutants (ETC3‐IB and ETC4‐IB) (Fig. 4B). Considering all mutants, there was no clear correlation between ATP contents and 2,3‐BDO production (Fig. 4D).

All ETC mutants harbouring isobutanol pathway exhibited increased NADH/NAD^+^ ratios compared to that of the parental strain. Approximately 1.1–2.5‐fold enhanced redox ratios were observed in ETC mutants, among which ETC4‐IB showed the highest redox ratio (Fig. 4E). As the expression of ubiquinone synthesis pathway was weakened by introducing synthetic UTRs (UbiE117, UbiE86 and UbiE26), gradually increased redox ratios were observed in mutants (Fig. 4E). Furthermore, highly increased redox ratios were observed in ETC3‐IB and ETC4‐IB, meaning that Nuo deletion was more crucial for perturbing intracellular redox ratio than that of terminal oxidase inactivation in case of isobutanol production (Fig. 4E). A clear positive correlation was identified between redox ratios and isobutanol titres (Fig. 4G). However, ATP contents were not much changed among UbiE and ETC mutants (Fig. 4F), resulting in no significant correlation between ATP contents and isobutanol titres (Fig. 4H).

Even if ATP was not supplied from ETC, it can be supplemented through SLPHO such as glycolysis and acetate formation. This could be the reason for comparable or higher ATP content of several mutants than that of the parental strain (Fig. 4B,F). Moreover, higher amount of glucose consumption and acetate production were observed by ETC mutants, which demonstrated that SLPHO was the major energy forming pathway in ETC mutants. It has been reported that OXPHO is favourable in terms of ATP/substrate yield, but disadvantageous in ATP production rate and enzymatic cost (Chen and Nielsen, 2019; Basan *et al*., 2015). Therefore, it is speculated that the formation of intracellular ATP was determined by much more complicated factors in ETC mutants.

### Scaled‐up cultivation to verify oxygen‐independent metabolism in ETC mutants

One of the challenging issues in industrial fermentation is the limitation of oxygen transfer in scaled‐up fermenters (Garcia‐Ochoa and Gomez, 2009). For instance, respiring cells could be present in oxygen‐rich spot and fermenting cell in oxygen‐limited spot (Chubukov *et al*., 2016). In other words, various phenotypes could be existing according to the oxygen level in different spots of a batch cultivation. The heterogeneous conditions in the fermenter can cause problems such as retardation of growth, generation of by‐products and reduction of product yield. For this reason, the problems of oxygen transfer in large‐scale fermentation can be resolved by developing strains carrying out cellular metabolism through oxygen‐insensitive way. It can be hypothesized that ETC mutants are less affected by extracellular oxygen concentration than the parental strain. To verify this, scaled‐up cultivation was implemented in various agitation conditions. The growth and extracellular metabolites profile of DSM01‐BDO and ETC4‐BDO were compared in 3 l‐scale fermentation because ETC4‐BDO exhibited the most significant improved 2,3‐BDO production compared with parental strain in flask experiments.

**Fig. 5:**
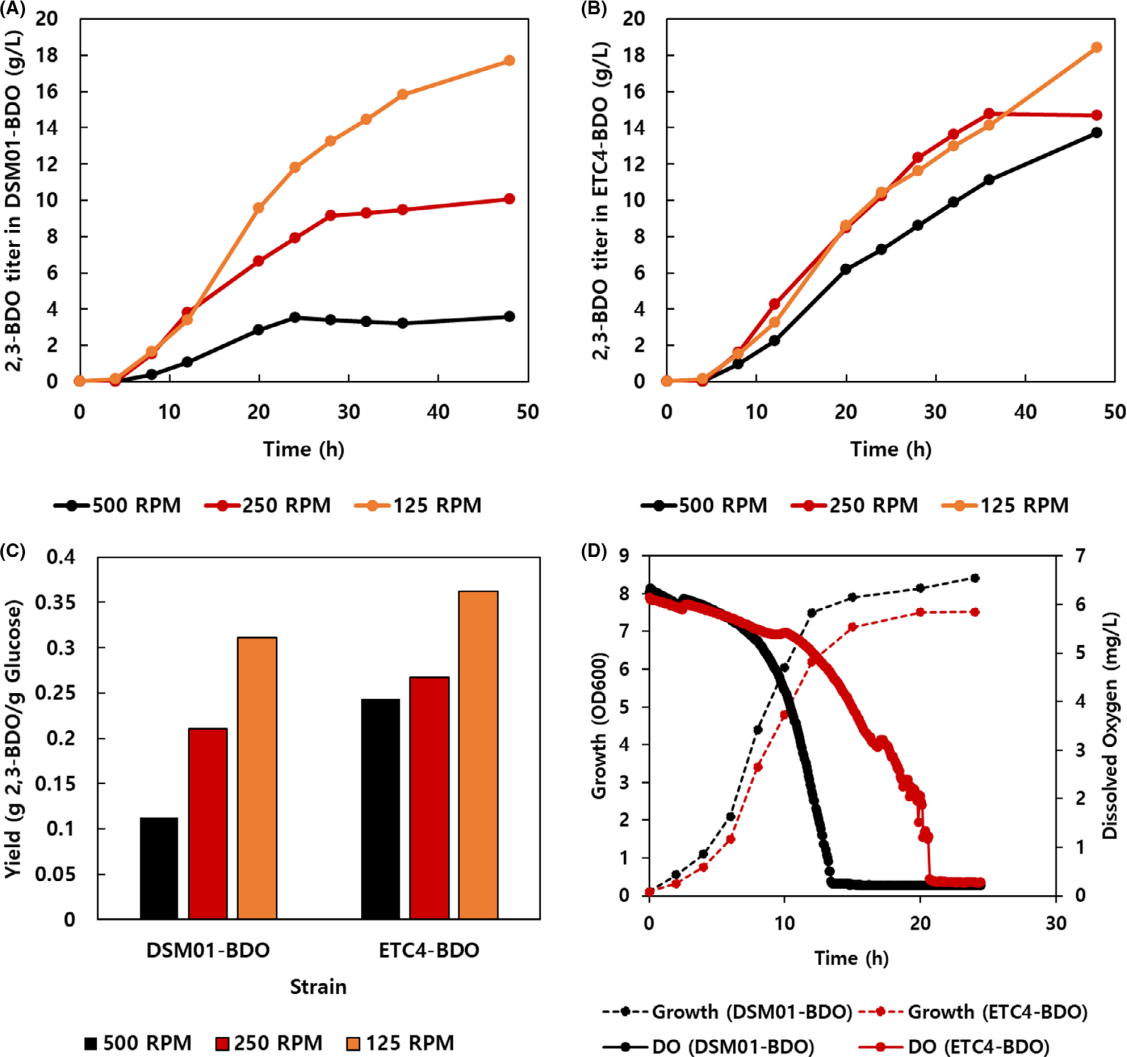
Comparison of DSM01‐BDO and ETC4‐BDO in large‐scale fermentation. The production titres of 2,3‐butanediol in (A) DSM01‐BDO and (B) ETC4‐BDO for 48 h fermentation are shown. (C) The yield of 2,3‐BDO in DSM01 and ETC4‐BDO were described. (D) The growth and dissolved oxygen (DO) were measured in DSM01‐BDO and ETC4‐BDO cultivations. The specific DO change rate was calculated by specific growth rate (*μ*) and DO change (Y_DO/Biomass_).

When cultivated in 125, 250 and 500 rpm, both of DSM01‐BDO and ETC4‐BDO produced less 2,3‐BDO as the agitation increased. Interestingly, the least oxygen‐induced effect was identified in ETC4‐BDO (Fig. 5A,B). The two strains produced comparable titres of 2,3‐BDO at oxygen‐limited condition (125 rpm). DSM01‐BDO produced 74% reduced amount of 2,3‐BDO at high agitation condition (500 rpm) compared with the amount of 2,3‐BDO by ETC4‐BDO. The 2,3‐BDO yield of DSM01‐BDO decreased by 64% but that of ETC4‐BDO reduced by 33% as the agitation increased from 125 to 500 rpm (Fig. 5C). Thus, it is confirmed that the ETC mutants can operate fermentative metabolism independent of oxygen supply.

It was also hypothesized that ETC mutants could have lower oxygen utilization than the parental strain. The specific dissolved oxygen (DO) change rate was calculated as previously published protocols (Long and Antoniewicz, 2019). The specific growth rate (μ) and specific DO change (YDO/Biomass) were measured during early and late exponential phase. The μ of DSM01‐BDO and ETC4‐BDO were 0.347 and 0.375 h^−1^, respectively, while the Y_DO/Biomass_ values were −0.613 mg (g dried cell weight (DCW))^−1^ and −0.424 mg gDCW^−1^, respectively, in exponential phase (4–8 h) (Fig. 5D). The calculated specific DO change rates (μ × Y_DO/Biomass_) of DSM01‐BDO and ETC4‐BDO were −0.212 and −0.159 mg gDCW^−1^ h^−1^, respectively (Table S2). This indicated that ETC4‐BDO exhibited 26% lower oxygen utilization compared to DSM01‐BDO in exponential phase. The difference of specific DO change rates between two strains was even greater in late exponential phase (8–12 h) (Table S2). Considering the specific DO change rate, ETC mutant clearly displayed lower oxygen utilization phenotype than the parental strain.

Although the oxygen‐insensitive phenotype appeared to have improved production of reduced compounds, ETC4‐BDO exhibited inferior final OD and higher glucose uptake, inducing larger amount of acetate than DSM01‐BDO (Fig. S3). The metabolic alteration could be due to energy deficiency and the activation of SLPHO to replenish ATP. The energy deficiency originating from the inactivation of ETC was compensated by increased glycolysis and acetate formation. The accumulation of acetate can cause acidification of culture broth and reduced product yields. In order to relieve acetate over‐production in ETC mutants, the alternative ATP supplementation can be applied. Several alternative ATP generating pathways are demonstrated in a previous report (Unden and Bongaerts, 1997). Furthermore, light or electric energy can be transformed to cellular energy through bacterial rhodopsin and electro‐fermentation techniques (Walter *et al*., 2007; Wu *et al*., 2019). Further studies utilizing external energy sources can be helpful to resolve the problems related to growth retardation and acetate accumulation in ETC mutants. It has been also documented that the inner membrane space is filled with various membrane proteins such as transporters and components of the respiration machinery (Bernsel and Daley, 2009; Papanastasiou *et al*., 2013). Thus, the removal of ETC components, which secures considerable volume of the membrane, could increase allocation of sugar transporters in the inner membrane space (Szenk *et al*., 2017). Therefore, for a comprehensive understanding of the physiology of the ETC mutants, an investigation of the redox and energy metabolism as well as changes of membrane proteome caused by the removal of ETC components are required.

## Conclusion

This report described the phenotypical traits (growth, metabolites, and oxygen uptake) and intracellular state (NADH and ATP) in ETC mutants. Because the ETC is engaged in the regeneration of redox cofactors, the attenuation of its activity affected the intracellular redox ratio. The metabolic perturbations in ETC mutants led to improved production of reduced metabolites, 2,3‐BDO and isobutanol and positive correlation was observed between redox ratios and production of reduced metabolites in several ETC mutants. The highest production of reduced metabolites was observed with ETC4 (*△nuo △cyo*) mutant. This result suggests that optimization of cellular metabolism can be obtained by modulating the ETC of the host strain. Furthermore, ETC mutants are more reliant on SLPHO than OXPHO compared to parental strain, which resulted in ETC mutants having oxygen‐independent metabolism. In conclusion, the discipline has potential for innovative engineering in the future, such as introduction of novel ETC components or efficient ATP generating module beyond innate ETC in *E. coli*.

## Experimental procedures

### Construction of strains and plasmids

All the strains and plasmids used in this study are listed in Table 1 and the oligomers (Bionics, Seoul, South Korea) are listed in Table S1. The genetic engineering for removal of ETC genes was based on λ‐red homologous recombination. The mutants were constructed in two types of NADH dehydrogenase and terminal oxidase in a combinatorial way which resulted in four ETC mutants. The 2,3‐butanediol synthesis pathway (*budABC* operon from *K. pneumoniae*) was inserted to pZS21 MCS vector which is called pZS‐BDO. The isobutanol pathway genes, namely, *ilvDC and budB* from *K. pneumonia* along with *kivD* and *adhA* from *L. lactis*, were inserted into pZA31 MCS and pBTBX‐2 vector, respectively, which were called pZA‐DCB and pBT‐DA. pZS‐BDO, pZA‐DCB, and pBT‐DA (Jung *et al*., 2017) were introduced into ETC mutants (Fig. 1).

In order to modulate the quinone synthesis in *E. coli*, genome editing was carried out using CRISPR/Cas9 system. The strains for genome editing were cultivated and prepared for electroporation. The 5′‐UTR sequence of demethylmenaquinone methyltransferase (*ubiE*) was targeted by crRNA. The crRNA and tracrRNA containing vector (pZS CRISPR *ubiE* RBS SacB), Cas9 harbouring vector (pCas9), and linear DNA fragment for DNA repair were introduced to parental strain for targeting, cleavage, and modifying ribosome binding sequence of *ubiE*. Then, artificial 5′UTR sequence was inserted by homologous recombination for recovery of cleavaged genomic DNA (Heo *et al*., 2017). The artificial UTR sequence was designed by ‘UTR designer’ to reduce the expression of *ubiE*. To confirm the mutations, the modified sequences were PCR amplified and analysed (Bionics, Seoul, Korea).

### Medium and cultivation

Lysogeny broth (LB; 5 g l^−1^ yeast extract, 10 g l^−1^ tryptone, 10 g l^−1^ NaCl) was utilized for all the genetic engineering procedures. Appropriate concentration of antibiotics (100 µg ml^−1^ of carbenicillin, 50 µg ml^−1^ of kanamycin and 34 µg ml^−1^ of chloramphenicol) were applied for selection of transformed strains with antibiotics resistance marker. During flask cultivation of strains containing two or more plasmids, half the concentration of antibiotics was applied to the cultivation medium. For preparation of 2,3‐BDO production medium, the modified M9 minimal medium (6 g l^−1^ Na_2_HPO_4_, 3 g l^−1^ KH_2_PO_4_, 1 g l^−1^ NH_4_Cl, 0.5 g l^−1^ NaCl, 0.01% of Thiamine‐HCl) also including 20 g l^−1^ of glucose, 2 g l^−1^ of yeast extract and 1 ml of trace elements (2.86 g l^−1^ H_3_BO_3_, 1.81 g l^−1^ MnCl_2_·4H_2_O, 0.22 g l^−1^ ZnSO_4_·7H_2_O, 0.39 g l^−1^ Na_2_MoO_4_·2H_2_O, 0.079 g l^−1^ CuSO_4_·5H_2_O, 49.4 mg l^−1^ Co(NO_3_)_2_·6H_2_O, and 0.9 g l^−1^ FeCl_3_·6H_2_O) per litre, was employed for flask cultivation. For preparation of isobutanol production medium, modified 2 × M9 medium (12 g l^−1^ Na_2_HPO_4_, 6 g l^−1^ KH_2_PO_4_, 2 g l^−1^ NH_4_Cl, 1 g l^−1^ NaCl, 0.01% of Thiamine‐HCl) including 40 g l^−1^ of glucose was utilized with same concentration of yeast extract and trace elements. One gram of calcium carbonate (CaCO_3_) was supplied for prolonged cultivation in isobutanol production. The seed inoculum was prepared in 15 ml conical tube by overnight cultivation in production medium at 37°C All host strains were cultivated in 250 ml Erlenmeyer flasks with 40 ml of working volume, at 37°C, and shaking at 250 rpm, with initial OD_600_ around 0.05. All the chemical reagents were purchased from Sigma‐Aldrich (St. Louis, MO, USA), unless otherwise mentioned.

### Analytical methods

The cell growth was measured via turbidity of the fermentation broth at 600 nm (OD_600_) using a UV‐Vis spectrophotometer DU730 (Beckman Coulter, Caguas, Puerto Rico). The DCW was derived from the calculation that DCW = OD_600_ × 0.36. For the analysis of extracellular metabolites, culture broth was acquired by centrifugation using CF‐10 centrifuge (9000 *g*) (Daihan Scientific, Seoul, South Korea), followed by filtration (0.2 μm pore) (Whatman, Maidstone, UK), and then, the supernatant was analysed by high‐performance liquid chromatography (HPLC) with refractive index detector Waters 2414 (Waters, MA, USA), employed with a SH1011 column (Shodex, Tokyo, Japan). A diluted sulphuric acid solution (10 mM) was applied for the HPLC mobile phase with a flow rate of 0.6 ml min^−1^.

### Analysis of intracellular redox and energy state

To measure the intracellular NADH/NAD^+^ ratio, NAD/NADH‐GloTM Assay kit (Promega, WI, US) was utilized. Briefly, the cell broth was prepared by cultivating to exponential phase using production medium. The culture broth was mixed with DTAB solution for 5 min. For measuring NADH, 0.4 N of HCl was added to the reaction mix, whereas for measuring NAD^+^, nothing was added. The sample was heated to 60°C for 15 min and cooled to 25°C. The HCl/Trizma solution was added to the reaction mix for NADH, whereas Trizma solution was added to that for NAD^+^. The prepared solutions were mixed with detection reagent at a ratio of 1:1 (v/v) and incubated for approximately 30 min. The luminescence was measured using microplate reader Biotek Synergy H1 (Biotek, VT, USA). The same methods were applied for measuring NAD(H) standard solution. The NADH and NAD^+^ were quantified, and the ratio of them was calculated.

For measuring the intracellular ATP content, BacTiter‐GloTM kit was utilized (Promega, WI, USA). Briefly, the cell broth was prepared by flask culture. The cell broth in exponential phase was taken and washed using distilled water. The same volume of cell broth and reaction mix was resuspended together and rested for 5 min in ambient condition. The luminescence was measured using microplate reader Biotek Synergy H1 (Biotek). The same methods were applied for measuring the ATP standard solution.

### Large‐scale fermentation and dissolved oxygen measurement

The seed inoculum was prepared in 15 ml conical tube using production medium, in a 37°C shaking incubator, by overnight cultivation. The seed was transferred to fresh medium for flask cultivation. When the culture reached mid‐exponential phase, 50 ml of culture broth was again inoculated to 3 l‐scale fermenter system (CNS, Daejeon, South Korea) with working volume of 1 L. The production medium contains 60 g l^−1^ of glucose, 1 × M9 salts, and the same components of production medium in the section ‘medium and cultivation’. The temperature and pH (Broadley James, CA, USA) in fermenter were maintained 37°C and 6.5, respectively, and potassium hydroxide (KOH) was used as a base solution. The aeration was fixed to 1.5 vvm (air), and agitation was varied from 125 to 500 rpm. The dissolved oxygen (DO) concentration was measured every 4 min at 600 rpm and 1.5 vvm (air) by using DO probe (Broadley James, CA, USA) equipped in the fermenter. The initial DO value that is saturated by air flow was set as 100% before inoculation in fermenters and the DO value that is calibrated by 2 M of sodium sulphite (Na_2_SO_3_) was set as 0%. The DO (%) value was measured every 4 min as fermentation proceeded. The DO concentrations (mg l^−1^) of air‐saturated culture medium (100%) and 2 M sodium sulphite solution (0%) were measured by DO meter, Orion3‐Star Plus, (Thermo Scientific, MA, USA) in order to convert DO (%) to absolute DO concentration (mg l^−1^). The DO concentration (mg l^−1^) of air‐saturated culture medium was 6.61 mg l^−1^ and that of 2 M sodium sulphite solution was 0.21 mg l^−1^. The specific DO change rate was calibrated by multiplication of specific growth rate (μ) and DO change (Y_DO/Biomass_) in DSM01‐BDO and ETC4‐BDO (*△nuo △cyo*) strain (Long and Antoniewicz, 2019).

## Conflict of interest

The author declares no competing financial interest.

## Supporting information


**Table S1.** Oligomers used in this study.
**Table S2.** Specific growth rate, specific DO change and specific DO change rate in DSM01‐BDO and ETC4‐BDO
**Fig. S1.** Effects of ZnSO4 to growth, production and specific yield of 2,3‐butanediol and isobutanol. (A) The growth, (B) production titers and (C) specific yield of 2,3‐butanediol were exhibited along with the varied concentration of ZnSO_4_. The growth retardation and comparable 2,3‐butanediol production were observed as the addition of ZnSO_4_ was increased. Thus the specific yield of 2,3‐butanediol was improved by addition of ZnSO_4_ (N.D. means “Not Detected”). (D) The growth, (E) production titers and (F) specific yield of isobutanol were exhibited along with the varied concentration of ZnSO_4_. The growth retardation and enhanced isobutanol production were observed as the addition of ZnSO_4_ was increased. Thus the specific yield of isobutanol was improved by addition of ZnSO_4_.
**Fig. S2.** Sequencing confirmation of UbiE knock down mutants. The modulated 5`‐UTR sequence of *ubiE* were PCR amplified and analyzed to confirm the mutations.
**Fig. S3.** Large scale fermentation results of DSM01‐BDO and ETC4‐BDO along with variation of agitation. The growth profiles of DSM01‐BDO and ETC4‐BDO in (A) 125 RPM, (D) 250 RPM and (G) 500 RPM for 48 h fermentation. The higher maximal growth and growth differences between DSM01‐BDO and ETC4‐BDO were exhibited as the agitation was increased. The glucose consumption and metabolites profiles of ETC4‐BDO in (B) 125 RPM, (E) 250 RPM, (H) 500 RPM were displayed. The glucose consumption and metabolites profiles of DSM01‐BDO in (C) 125 RPM, (F) 250 RPM, (I) 500 RPM were displayed.

## References

[mbt213669-bib-0001] Abdel‐Hamid, A.M. , Attwood, M.M. , and Guest, J.R. (2001) Pyruvate oxidase contributes to the aerobic growth efficiency of *Escherichia coli* . Microbiol 147: 1483–1498. 10.1099/00221287-147-6-1483 11390679

[mbt213669-bib-0002] Baek, J.M. , Mazumdar, S. , Lee, S.W. , Jung, M.Y. , Lim, J.H. , Seo, S.W. , *et al.* (2013) Butyrate production in engineered Escherichia coli with synthetic scaffolds. Biotechnol Bioeng 139: 2790–2794. 10.1002/bit.24925 23568786

[mbt213669-bib-0003] Basan, M. , Hui, S. , Okano, H. , Zhang, Z. , Shen, Y. , Williamson, J.R. , and Hwa, T. (2015) Overflow metabolism in *Escherichia coli* results from efficient proteome allocation. Nature 528: 99–104. 26632588 10.1038/nature15765 PMC4843128

[mbt213669-bib-0004] van Beilen, J.W.A. , and Hellingwerf, K.J. (2016) All three endogenous Quinone species of *Escherichia coli* are involved in controlling the activity of the aerobic/anaerobic response regulator ArcA. Front Microbiol 7: 1339. 27656164 10.3389/fmicb.2016.01339 PMC5013052

[mbt213669-bib-0005] Bekker, M. , de Vries, S. , Ter Beek, A. , Hellingwerf, K.J. , and de Mattos, M.J.T. (2009) Respiration of *Escherichia coli* can be fully uncoupled via the nonelectrogenic terminal cytochrome bd‐II oxidase. J. Bacteriol 191: 5510–5517. 19542282 10.1128/JB.00562-09 PMC2725625

[mbt213669-bib-0006] Bernsel, A. , and Daley, D.O. (2009) Exploring the inner membrane proteome of *Escherichia coli*: which proteins are eluding detection and why? Trends Microbiol. 17: 444–449. 19766000 10.1016/j.tim.2009.07.005

[mbt213669-bib-0007] Bringaud, F. , Ebikeme, C. , and Boshart, M. (2010) Acetate and succinate production in amoebae, helminths, diplomonads, trichomonads and trypanosomatids: common and diverse metabolic strategies used by parasitic lower eukaryotes. Parasitology 137: 1315–1331. 20028611 10.1017/S0031182009991843

[mbt213669-bib-0008] Budin, I. , de Rond, T. , Chen, Y. , Chan, L.J.G. , Petzold, C.J. , and Keasling, J.D. (2018) Viscous control of cellular respiration by membrane lipid composition. Science 362: 1186–1189. 30361388 10.1126/science.aat7925

[mbt213669-bib-0009] Chan, S. , Jantama, S.S. , Kanchanatawee, S. , and Jantama, K. (2016) Process optimization on micro‐aeration supply for high production yield of 2,3‐butanediol from maltodextrin by metabolically‐engineered *Klebsiella oxytoca* . PLoS One 11: e0161503. 27603922 10.1371/journal.pone.0161503 PMC5014425

[mbt213669-bib-0010] Chen, X. , Alonso, A.P. , Allen, D.K. , Reed, J.L. , and Shachar‐Hill, Y. (2011) Synergy between (13)C‐metabolic flux analysis and flux balance analysis for understanding metabolic adaptation to anaerobiosis in *E. coli* . Metab Eng 13: 38–48. 21129495 10.1016/j.ymben.2010.11.004

[mbt213669-bib-0011] Chen, Y. , and Nielsen, J. (2019) Energy metabolism controls phenotypes by protein efficiency and allocation. Proc Natl Acad Sci USA 116: 17592–17597. 31405984 10.1073/pnas.1906569116 PMC6717264

[mbt213669-bib-0012] Chubukov, V. , Mukhopadhyay, A. , Petzold, C.J. , Keasling, J.D. , and Martin, H.G. (2016) Synthetic and systems biology for microbial production of commodity chemicals. NPJ Syst Biol Appl 2: 16009. 28725470 10.1038/npjsba.2016.9 PMC5516863

[mbt213669-bib-0013] Garcia‐Ochoa, F. , and Gomez, E. (2009) Bioreactor scale‐up and oxygen transfer rate in microbial processes: an overview. Biotechnol Adv 27: 153–176. 19041387 10.1016/j.biotechadv.2008.10.006

[mbt213669-bib-0014] Gonzalez, J.E. , Long, C.P. , and Antoniewicz, M.R. (2017) Comprehensive analysis of glucose and xylose metabolism in *Escherichia coli* under aerobic and anaerobic conditions by 13C metabolic flux analysis. Metab Eng 39: 9–18. 27840237 10.1016/j.ymben.2016.11.003 PMC5845445

[mbt213669-bib-0015] Goodwin, M.L. , Gladden, L.B. , Nijsten, M.W.N. , and Jones, K.B. (2015) Lactate and cancer: revisiting the warburg effect in an era of lactate shuttling. Front Nutr 1: 27. 25988127 10.3389/fnut.2014.00027 PMC4428352

[mbt213669-bib-0016] Heo, M.J. , Jung, H.M. , Um, J. , Lee, S.W. , and Oh, M.K. (2017) Controlling citrate synthase expression by CRISPR/Cas9 genome editing for n‐Butanol production in *Escherichia coli* . ACS Synth Biol 6: 182–189. 27700055 10.1021/acssynbio.6b00134

[mbt213669-bib-0017] Ji, X.‐J. , Huang, H. , and Ouyang, P.‐K. (2011) Microbial 2,3‐butanediol production: a state‐of‐the‐art review. Biotechnol Adv 29: 351–364. 21272631 10.1016/j.biotechadv.2011.01.007

[mbt213669-bib-0018] Jiang, W. , Bikard, D. , Cox, D. , Zhang, F. , and Marraffini, L.A. (2013) RNA‐guided editing of bacterial genomes using CRISPR‐Cas systems. Nat Biotechnol 31: 233–239. 23360965 10.1038/nbt.2508 PMC3748948

[mbt213669-bib-0019] Jung, H.M. , Im, D.K. , Lim, J.H. , Jung, G.Y. , and Oh, M.K. (2019) Metabolic perturbations in mutants of glucose transporters and their application in metabolite production in *Escherichia coli* . Microb Cell Fact 18: 170. 31601271 10.1186/s12934-019-1224-8 PMC6786474

[mbt213669-bib-0020] Jung, H.M. , Kim, Y.H. , and Oh, M.K. (2017) Formate and nitrate utilization in *Enterobacter aerogenes* for semi‐anaerobic production of isobutanol. Biotechnol J 12: 1700121. 10.1002/biot.201700121 28731532

[mbt213669-bib-0021] Jung, M.Y. , Mazumdar, S. , Shin, S. , Yang, K.S. , Lee, J. , and Oh, M.K. (2014) Improvement of 2,3‐butanediol yield in *Klebsiella pneumoniae* by deletion of the pyruvate formate‐lyase gene. Appl Environ Microbiol 80: 6195–6203. 25085487 10.1128/AEM.02069-14 PMC4178695

[mbt213669-bib-0022] Kalnenieks, U. , Balodite, E. , and Rutkis, R. (2019) Metabolic engineering of bacterial respiration: high vs. low P/O and the case of *Zymomonas mobilis* . Front Bioeng Biotechnol 7: 327. 31781557 10.3389/fbioe.2019.00327 PMC6861446

[mbt213669-bib-0023] Kim, Y. , Ingram, L.O. , and Shanmugam, K.T. (2008) Dihydrolipoamide dehydrogenase mutation alters the NADH sensitivity of pyruvate dehydrogenase complex of *Escherichia coli* K‐12. J Bacteriol 190: 3851–3858. 18375566 10.1128/JB.00104-08 PMC2395023

[mbt213669-bib-0024] Koch‐Koerfges, A. , Pfelzer, N. , Platzen, L. , Oldiges, M. , and Bott, M. (2013) Conversion of *Corynebacterium glutamicum* from an aerobic respiring to an aerobic fermenting bacterium by inactivation of the respiratory chain. Biochim Biophys Acta 1827: 699–708. 23416842 10.1016/j.bbabio.2013.02.004

[mbt213669-bib-0025] Korshunov, S. , and Imlay, J.A. (2006) Detection and quantification of superoxide formed within the periplasm of *Escherichia coli* . J Bacteriol 188: 6326–6334. 16923900 10.1128/JB.00554-06 PMC1595388

[mbt213669-bib-0026] Lee, J.H. , Lama, S. , Kim, J.R. , and Park, S.H. (2019) Production of 1,3‐propanediol from glucose by recombinant *Escherichia coli* BL21(DE3). Biotechnol BIoproc Eng 23: 250–258.

[mbt213669-bib-0027] Liu, Y. , Zhu, Y. , Ma, W. , Shin, H.D. , Li, J. , Liu, L. , *et al* (2014) Spatial modulation of key pathway enzymes by DNA‐guided scaffold system and respiration chain engineering for improved N‐acetylglucosamine production by *Bacillus subtilis* . Metab Eng 24: 61–69. 24815549 10.1016/j.ymben.2014.04.004

[mbt213669-bib-0028] Long, C.P. , and Antoniewicz, M.R. (2019) High‐resolution ^13^C metabolic flux analysis. Nat Protoc 14: 2856–2877. 31471597 10.1038/s41596-019-0204-0

[mbt213669-bib-0029] Matsushita, K. , Ohnishi, T. , and Kaback, H.R. (1987) NADH‐Ubiquinone oxidoreductases of the *Escherichia coli* aerobic respiratory chain. Biochemistry‐US 26: 7732–7737. 10.1021/bi00398a029 3122832

[mbt213669-bib-0030] Mazumdar, S. , Lee, J. , and Oh, M.‐K. (2013) Microbial production of 2,3 butanediol from seaweed hydrolysate using metabolically engineered *Escherichia coli* . Bioresource Technol 136: 329–336. 10.1016/j.biortech.2013.03.013 23567699

[mbt213669-bib-0031] Moreau, P.L. (2004) Diversion of the metabolic flux from pyruvate dehydrogenase to pyruvate oxidase decreases oxidative stress during glucose metabolism in nongrowing *Escherichia coli* cells incubated under aerobic, phosphate starvation conditions. J Bacteriol 186: 7364–7368. 15489448 10.1128/JB.186.21.7364-7368.2004 PMC523199

[mbt213669-bib-0032] Nies, S.C. , Dinger, R. , Chen, Y. , Wordofa, G.G. , Kristensen, M. , Schneider, K. , *et al* (2019) The metabolic response of Pseudomonas taiwanensis to NADH dehydrogenase deficiency. *bioRxiv* 624536.

[mbt213669-bib-0033] Papanastasiou, M. , Orfanoudaki, G. , Koukaki, M. , Kountourakis, N. , Sardis, M.F. , Aivaliotis, M. , *et al* (2013) The *Escherichia coli* peripheral inner membrane proteome. Mol Cell Proteom 12: 599–610. 10.1074/mcp.M112.024711 PMC3591654 23230279

[mbt213669-bib-0034] Pfeiffer, T. , and Morley, A. (2014) An evolutionary perspective on the Crabtree effect. Front Mol Biosci 1: 17. 25988158 10.3389/fmolb.2014.00017 PMC4429655

[mbt213669-bib-0035] Prüss, B.M. , Nelms, J.M. , Park, C. , and Wolfe, A.J. (1994) Mutations in NADH:ubiquinone oxidoreductase of *Escherichia coli* affect growth on mixed amino acids. J Bacteriol 176: 2143–2150. 8157582 10.1128/jb.176.8.2143-2150.1994 PMC205332

[mbt213669-bib-0036] Puustinen, A. , Finel, M. , Haltia, T. , Gennis, R.B. , and Wikstrom, M. (1991) Properties of the two terminal oxidases of *Escherichia coli* . Biochemistry‐US 30: 3936–3942. 10.1021/bi00230a019 1850294

[mbt213669-bib-0037] Ravcheev, D.A. , and Thiele, I. (2016) Genomic analysis of the human gut microbiome suggests novel enzymes involved in quinone biosynthesis. Front Microbiol 7: 128. 26904004 10.3389/fmicb.2016.00128 PMC4746308

[mbt213669-bib-0038] Schulte, M. , Mattay, D. , Kriegel, S. , Hellwig, P. , and Friedrich, T. (2014) Inhibition of *Escherichia coli* respiratory complex I by Zn(2^+^). Biochemistry‐US 53: 6332–6339. 10.1021/bi5009276 25238255

[mbt213669-bib-0039] Sousa, P.M.F. , Videira, M.A.M. , Bohn, A. , Hood, B.L. , Conrads, T.P. , Goulao, L.F. , and Melo, A.M.P. (2012) The aerobic respiratory chain of *Escherichia coli*: from genes to supercomplexes. Microbiol‐Sgm 158: 2408–2418. 10.1099/mic.0.056531-0 22700653

[mbt213669-bib-0040] Szenk, M. , Dill, K.A. , and de Graff, A.M.R. (2017) Why do fast growing bacteria enter overflow metabolism? Testing the membrane real estate hypothesis. Cell Syst 5: 95–104. 28755958 10.1016/j.cels.2017.06.005

[mbt213669-bib-0041] Unden, G. , and Bongaerts, J. (1997) Alternative respiratory pathways of *Escherichia coli*: energetics and transcriptional regulation in response to electron acceptors. Biochim Biophys Acta 1320: 217–234. 9230919 10.1016/s0005-2728(97)00034-0

[mbt213669-bib-0042] Walter, J.M. , Greenfield, D. , Bustamante, C. , and Liphardt, J. (2007) Light‐powering *Escherichia coli* with proteorhodopsin. Proc Natl Acad Sci USA 104: 2408–2412. 17277079 10.1073/pnas.0611035104 PMC1892948

[mbt213669-bib-0043] Ward, B. (2015) Chapter 11 ‐ Bacterial Energy Metabolism In Molecular medical microbiology, 2nd edn Tang, Y.‐W. , Sussman, M. , Liu, D. , Poxton, I. , and Schwartzman, J. (eds). Boston, MA, USA: Academic Press, pp. 201–233.

[mbt213669-bib-0044] Wong, M.S. , Li, M. , Black, R.W. , Le, T.Q. , Puthli, S. , Campbell, P. , and Monticello, D.J. (2014) Microaerobic conversion of glycerol to ethanol in *Escherichia coli* . Appl Environ Microb 80: 3276–3282. 10.1128/AEM.03863-13 PMC4018910 24584248

[mbt213669-bib-0045] Wu, H. , Tuli, L. , Bennett, G.N. , and San, K.Y. (2015) Metabolic transistor strategy for controlling electron transfer chain activity in *Escherichia coli* . Metab Eng 28: 159–168. 25596510 10.1016/j.ymben.2015.01.002 PMC4355220

[mbt213669-bib-0046] Wu, Z. , Wang, J. , Liu, J. , Wang, Y. , Bi, C. , and Zhang, X. (2019) Engineering an electroactive *Escherichia coli* for the microbial electrosynthesis of succinate from glucose and CO_2_ . Microb Cell Fact 18: 15. 30691454 10.1186/s12934-019-1067-3 PMC6348651

[mbt213669-bib-0047] Wu, P. , Wang, G. , Wang, G. , Børresen, B.T. , Liu, H. , and Zhang, J. (2016) Butanol production under microaerobic conditions with a symbiotic system of *Clostridium acetobutylicum* and *Bacillus cereus* . Microb Cell Fact 15: 8. 26762531 10.1186/s12934-016-0412-z PMC4712489

[mbt213669-bib-0048] Zamboni, N. , Mouncey, N. , Hohmann, H.P. , and Sauer, U. (2003) Reducing maintenance metabolism by metabolic engineering of respiration improves riboflavin production by *Bacillus subtilis* . Metab Eng 5: 49–55. 12749844 10.1016/s1096-7176(03)00007-7

[mbt213669-bib-0049] Zambrano, M.M. , and Kolter, R. (1993) *Escherichia coli* mutants lacking NADH dehydrogenase I have a competitive disadvantage in stationary phase. J Bacteriol 175: 5642–5647. 8366049 10.1128/jb.175.17.5642-5647.1993 PMC206622

[mbt213669-bib-0050] Zhang, L. , Bao, W. , Wei, R. , Fu, S. , and Gong, H. (2018) Inactivating NADH:quinone oxidoreductases affects the growth and metabolism of *Klebsiella pneumoniae* . Biotechnol Appl Biochem 65: 857–864. 30063071 10.1002/bab.1684

[mbt213669-bib-0051] Zhu, J. , Sánchez, A. , Bennett, G.N. , and San, K.‐Y. (2011) Manipulating respiratory levels in *Escherichia coli* for aerobic formation of reduced chemical products. Metab Eng 13: 704–712. 22001430 10.1016/j.ymben.2011.09.006 PMC3210874

