# Supplementary material for: Improved production of 2,3‐butanediol and isobutanol by engineering electron transport chain in Escherichia coli
Source: Microb Biotechnol. 2020 Sep 20;14(1):213–26. doi: 10.1111/1751-7915.13669 (PMC7888471; doi:10.1111/1751-7915.13669)

Supporting information

Improved production of 2,3-butanediol and isobutanol by engineering electron transport chain in *Escherichia coli*

Authors

Hwi-Min Jung^a^, Jae-Ho Han^a^, Min-Kyu Oh^a^*

Affiliations

^a^Department of Chemical and Biological Engineering, Korea University, 145 Anam-ro, Seongbuk-gu, Seoul 02841, Korea

* Corresponding author: Min-Kyu Oh ([mkoh@korea.ac.kr](mailto:mkoh@korea.ac.kr)).

Author E-mail address

Hwi-Min Jung ([jhm1215@korea.ac.kr](mailto:jhm1215@korea.ac.kr))

Jae-Ho Han ([maal97@nate.com](mailto:maal97@nate.com))

Min-Kyu Oh ([mkoh@korea.ac.kr](mailto:mkoh@korea.ac.kr))

Tel : +82-2-3290-3308

Table S1 Oligomers used in this study

The homologous sequences were written in capital and annealing sequence for PCR amplification were presented in small letters for gene deletion oligomers.

| Name | Sequence (5` to 3`) | Description |
| --- | --- | --- |
| Ndh FKF Fwd | AAAAGATTGTGATTGTCGGCGGCGGTGCTGGTGGGCTGGAAATGGCAACAgtgtaggctggagctgcttc | Ndh deletion |
| Ndh FKF Rev | CAGGTTACCCATCAGGCTACCGACGGTGGAGAAGTTCGACAGCGATACCAgtccatatgaatatcctcct | Ndh deletion |
| Ndh Conf Fwd | tgcgcttcttatcaggccta | Ndh deletion |
| Ndh Conf Rev | ccaatcatgcaagcgaagga | Ndh deletion |
| Nuo FKF Fwd | GTGCTGGGTATTCCGGCAAGCGACGTCGAAGGTGTGGCAACGTTCTACAGgtgtaggctggagctgcttc | Nuo deletion |
| Nuo FKF Rev | CAAACGGCACTTGCGAACGGTGCGCAGTCGGCTGGTTGTTACCTTCCATCgtccatatgaatatcctcct | Nuo deletion |
| Nuo Conf Fwd | ccacaaaccgaggcttttga | Nuo deletion |
| Nuo Conf Rev | gtcagtccttcggcgatttc | Nuo deletion |
| Cyd FKF Fwd | ATTGCGTTTTATCTGGTGGCTGCTGGTTGGCGTTCTGCTGATTGGTTTTGgtgtaggctggagctgcttc | Cyd deletion |
| Cyd FKF Rev | GCTCCTTACTTAGTACAGAGAGTGGGTGTTACGTTCAATATCTTCTTTGGgtccatatgaatatcctcct | Cyd deletion |
| Cyd Conf Fwd | gccgtggattgctgtagaag | Cyd deletion |
| Cyd Conf Rev | cggcccacatcagcaataaa | Cyd deletion |
| Cyo FKF Fwd | GCTGGCATTATTTTGGGAGGTCTGGCGCTCGTTGGCCTGATCACTTACTTgtgtaggctggagctgcttc | Cyo deletion |
| Cyo FKF Rev | GCATCGCAGAATGGAACACGATGCGGCCCTGATACATGGTGAACAGCCAGgtccatatgaatatcctcct | Cyo deletion |
| Cyo Conf Fwd | acacgaaggtatggaaggca | Cyo deletion |
| Cyo Conf Rev | caatgacgatacctgcaccg | Cyo deletion |
| Cbd FKF Fwd | AACATTGCGCTTCATCTGGTGGCTGCTGATTGGCGTGATCCTGGTGGTCTgtgtaggctggagctgcttc | Cbd deletion |
| Cbd FKF Rev | CCAGAGAGTGTAGAGCAACACAATGGGCAAAAATATCAGCACGATTACCAgtccatatgaatatcctcct | Cbd deletion |
| Cbd Conf Fwd | aaatggcgctctggagtttg | Cbd deletion |
| Cbd Conf Rev | caccacactttccagcttca | Cbd deletion |
| UbiE crRNA Sen | AAACTTACACTTCTGGAACAATTTTTTGATGAGCG | UbiE RBS targeting crRNA cloning |
| UbiE crRNA AtSen | AAAACGCTCATCAAAAAATTGTTCCAGAAGTGTAA | UbiE RBS targeting crRNA cloning |
| UbiE Conf Fwd | ggaacgtggcggtcgaaa | UbiE RBS modification confirmation |
| UbiE Conf Rev | ctgcaatcaatcgtgaatcg | UbiE RBS modification confirmation |
| UbiE117 Rescue AtSen | GCGACGGTCTGAAAACCAAAGTGCGTCGTTTCTTGTGACTTATCCACCATTTTTGTTTCTCGTTTCGACCGCCACGTTCCAG | UbiE RBS modification |
| UbiE86 Rescue AtSen | GCGACGGTCTGAAAACCAAAGTGCGTCGTTTCTTGTGACTTATCCACCATTTTTGTTCCTAGTTTCGACCGCCACGTTCCAG | UbiE RBS modification |
| UbiE26 Rescue AtSen | GCGACGGTCTGAAAACCAAAGTGCGTCGTTTCTTGTGACTTATCCACCATTTTTGTTTCTAATTTCGACCGCCACGTTCCAG | UbiE RBS modification |
| UbiE Rescue Sen Univ | CCGGGTAGAAATCTAGGGCATCGACGCCCAATCTGTTACACTTCTGGAACGTGGCGGTCGAAA | UbiE RBS modification |
| UbiE RBS SeqPCR Fwd | AGTCGCGCTAGCCCATTG | PCR amplification for UbiE RBS sequencing |
| UbiE RBS SeqPCR Rev | gttacgcagaccaaacgaaa | PCR amplification for UbiE RBS sequencing |
| UbiE RBS Seq | atcaatcgtgaatcgcttcc | UbiE RBS sequencing |

Table S2 Specific growth rate, specific DO change and specific DO change rate in DSM01-BDO and ETC4-BDO

Early exponential phase (4~8h)

|  | **DSM01-BDO** | **ETC4-BDO** | **Unit** |
| --- | --- | --- | --- |
| **μ** | **0.347** | **0.375** | **h^-1^** |
| **Y_DO/Biomass_** | **-0.613** | **-0.424** | **mg (g DCW)^-1^** |
| **Specific DO change rate** | **-0.212** | **-0.159** | **mg (g DCW)^-1^ h^-1^** |

Late exponential phase (8~12h)

|  | **DSM01-BDO** | **ETC4-BDO** | **Unit** |
| --- | --- | --- | --- |
| **μ** | **0.133** | **0.149** | **h^-1^** |
| **Y_DO/Biomass_** | **-2.759** | **-0.460** | **mg (g DCW)^-1^** |
| **Specific DO change rate** | **-0.368** | **-0.069** | **mg (g DCW)^-1^ h^-1^** |

Figure S1 Effects of ZnSO4 to growth, production and specific yield of 2,3-butanediol and isobutanol

(A) The growth, (B) production titers and (C) specific yield of 2,3-butanediol were exhibited along with the varied concentration of ZnSO_4_. The growth retardation and comparable 2,3-butanediol production were observed as the addition of ZnSO_4_ was increased. Thus the specific yield of 2,3-butanediol was improved by addition of ZnSO_4_ (N.D. means “Not Detected”). (D) The growth, (E) production titers and (F) specific yield of isobutanol were exhibited along with the varied concentration of ZnSO_4_. The growth retardation and enhanced isobutanol production were observed as the addition of ZnSO_4_ was increased. Thus the specific yield of isobutanol was improved by addition of ZnSO_4_.


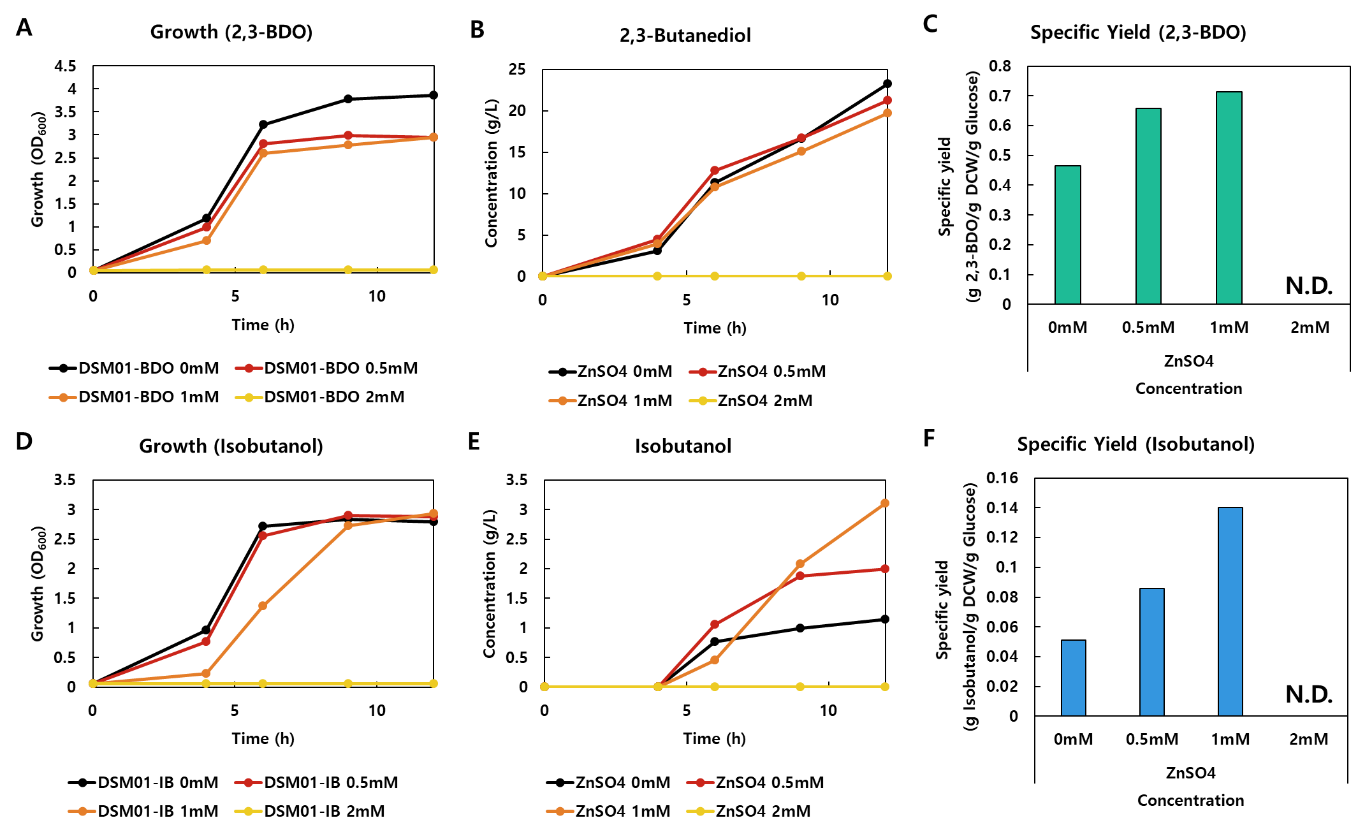


Figure S2 Sequencing confirmation of UbiE knock down mutants

The modulated 5`-UTR sequence of *ubiE* were PCR amplified and analyzed to confirm the mutations.


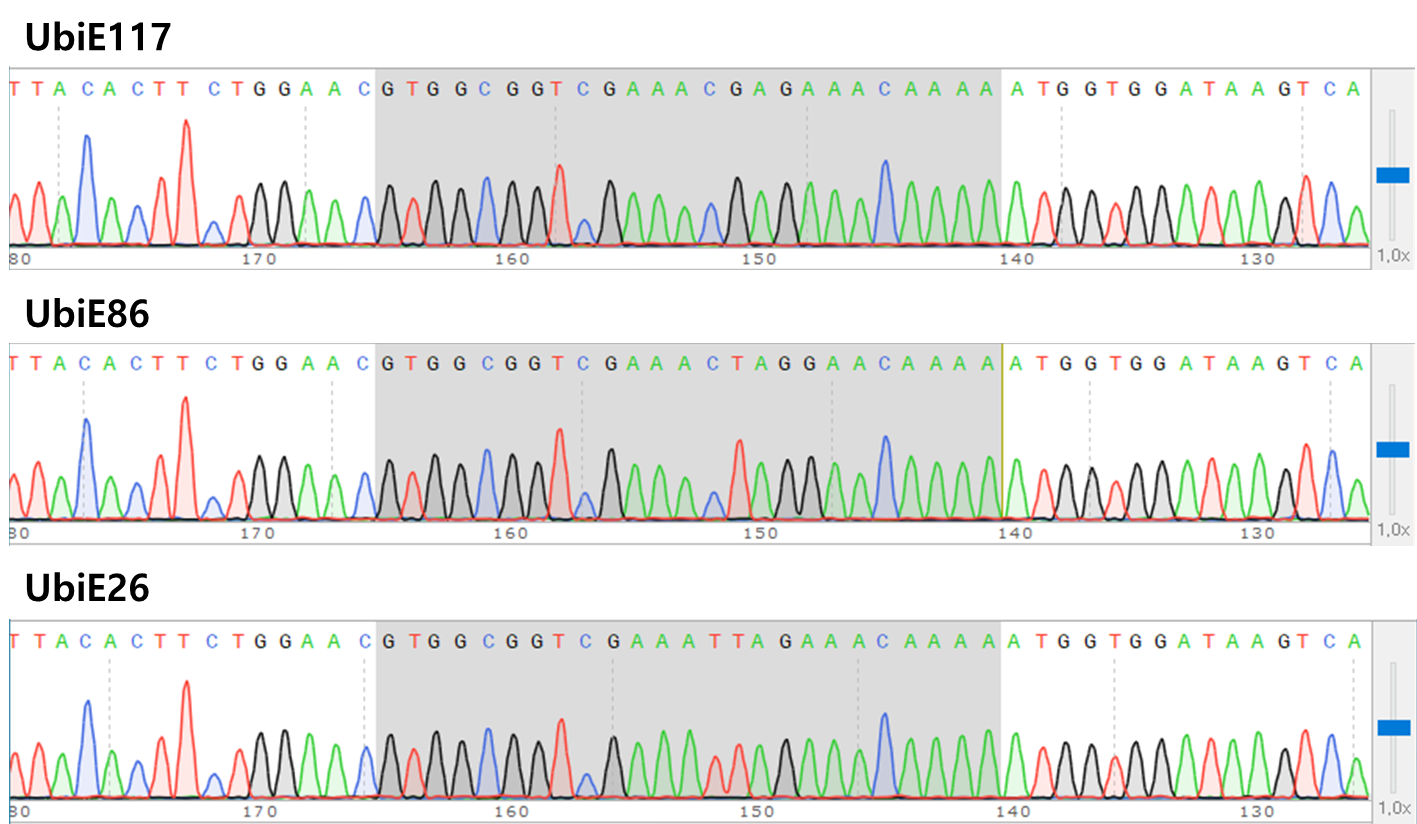


Figure S3 Large scale fermentation results of DSM01-BDO and ETC4-BDO along with variation of agitation.

The growth profiles of DSM01-BDO and ETC4-BDO in (A) 125 RPM, (D) 250 RPM and (G) 500 RPM for 48 h fermentation. The higher maximal growth and growth differences between DSM01-BDO and ETC4-BDO were exhibited as the agitation was increased. The glucose consumption and metabolites profiles of ETC4-BDO in (B) 125 RPM, (E) 250 RPM, (H) 500 RPM were displayed. The glucose consumption and metabolites profiles of DSM01-BDO in (C) 125 RPM, (F) 250 RPM, (I) 500 RPM were displayed.


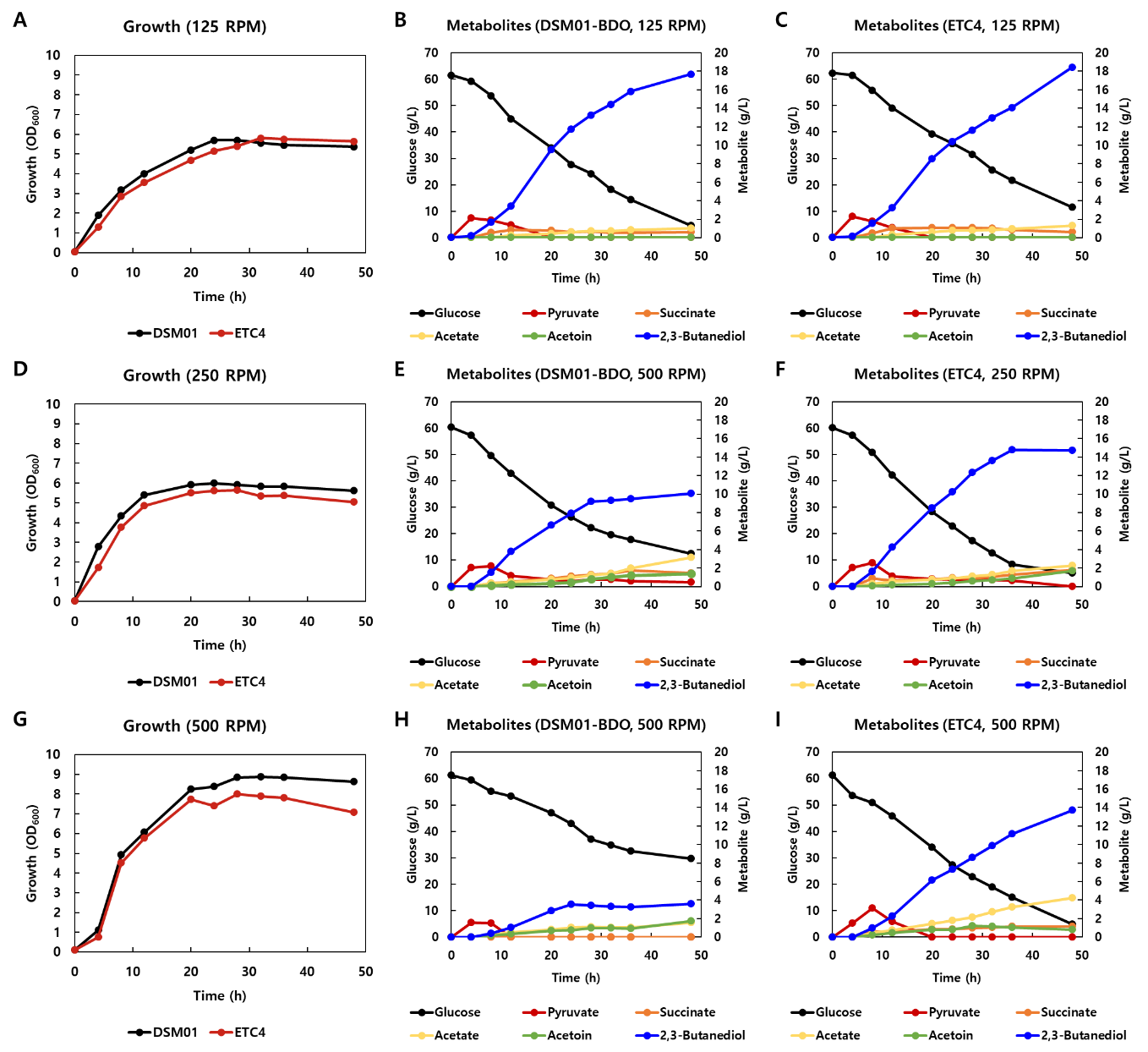

Supplement: Supplementary file 1 — Table S1. Oligomers used in this study. Table S2. Specific growth rate, specific DO change and specific DO change rate in DSM01‐BDO and ETC4‐BDO Fig. S1. Effects of ZnSO4 to growth, production and specific yield of 2,3‐butanediol and isobutanol. (A) The growth, (B) production titers and (C) specific yield of 2,3‐butanediol were exhibited along with the varied concentration of ZnSO4. The growth retardation and comparable 2,3‐butanediol production were observed as the addition of ZnSO4 was increased. Thus the specific yield of 2,3‐butanediol was improved by addition of ZnSO4 (N.D. means “Not Detected”). (D) The growth, (E) production titers and (F) specific yield of isobutanol were exhibited along with the varied concentration of ZnSO4. The growth retardation and enhanced isobutanol production were observed as the addition of ZnSO4 was increased. Thus the specific yield of isobutanol was improved by addition of ZnSO4. Fig. S2. Sequencing confirmation of UbiE knock down mutants. The modulated 5`‐UTR sequence of ubiE were PCR amplified and analyzed to confirm the mutations. Fig. S3. Large scale fermentation results of DSM01‐BDO and ETC4‐BDO along with variation of agitation. The growth profiles of DSM01‐BDO and ETC4‐BDO in (A) 125 RPM, (D) 250 RPM and (G) 500 RPM for 48 h fermentation. The higher maximal growth and growth differences between DSM01‐BDO and ETC4‐BDO were exhibited as the agitation was increased. The glucose consumption and metabolites profiles of ETC4‐BDO in (B) 125 RPM, (E) 250 RPM, (H) 500 RPM were displayed. The glucose consumption and metabolites profiles of DSM01‐BDO in (C) 125 RPM, (F) 250 RPM, (I) 500 RPM were displayed. [file MBT2-14-213-s001.docx]
